# Andrographolide, a Novel NF-**κ**B Inhibitor, Induces Vascular Smooth Muscle Cell Apoptosis via a Ceramide-p47phox-ROS Signaling Cascade

**DOI:** 10.1155/2013/821813

**Published:** 2013-12-29

**Authors:** Yu-Ying Chen, Ming-Jen Hsu, Joen-Rong Sheu, Lin-Wen Lee, Cheng-Ying Hsieh

**Affiliations:** ^1^Graduate Institute of Medical Sciences, School of Medicine, Taipei Medical University, 250 Wu-Hsing Street, Taipei 11031, Taiwan; ^2^Department of Pharmacology, School of Medicine, Taipei Medical University, 250 Wu-Hsing Street, Taipei 11031, Taiwan; ^3^Department of Microbiology and Immunology, Taipei Medical University, 250 Wu-Hsing Street, Taipei 11031, Taiwan

## Abstract

Atherosclerosis is linked with the development of many cardiovascular complications. Abnormal proliferation of vascular smooth muscle cells (VSMCs) plays a crucial role in the development of atherosclerosis. Accordingly, the apoptosis of VSMCs, which occurs in the progression of vascular proliferation, may provide a beneficial strategy for managing cardiovascular diseases. Andrographolide, a novel nuclear factor-**κ**B inhibitor, is the most active and critical constituent isolated from the leaves of *Andrographis paniculata*. Recent studies have indicated that andrographolide is a potential therapeutic agent for treating cancer through the induction of apoptosis. In this study, the apoptosis-inducing activity and mechanisms in andrographolide-treated rat VSMCs were characterized. Andrographolide significantly induced reactive oxygen species (ROS) formation, p53 activation, Bax, and active caspase-3 expression, and these phenomena were suppressed by pretreating the cells with N-acetyl-L-cysteine, a ROS scavenger, or diphenylene iodonium, a nicotinamide adenine dinucleotide phosphate (NADPH) oxidase (Nox) inhibitor. Furthermore, p47phox, a Nox subunit protein, was phosphorylated in andrographolide-treated rat VSMCs. However, pretreatment with 3-O-methyl-sphingomyelin, a neutral sphingomyelinase inhibitor, significantly inhibited andrographolide-induced p47phox phosphorylation as well as Bax and active caspase-3 expression. Our results collectively demonstrate that andrographolide-reduced cell viability can be attributed to apoptosis in VSMCs, and this apoptosis-inducing activity was associated with the ceramide-p47phox-ROS signaling cascade.

## 1. Introduction

Atherosclerosis remains a major and increasing health concern in developed countries, although prevention strategies have substantially increased. Consequently, developing novel therapeutic agents for atherosclerosis patients remains a major research priority [1]. Aberrant vascular smooth muscle cells (VSMCs) proliferation has been shown to play a critical role in the pathogenesis of atherosclerosis-related events including in-stent restenosis, restenosis after percutaneous transluminal angioplasty, transplant vasculopathy, and vein bypass graft failure [2, 3]. Therefore, inhibition of VSMC proliferation might be a major target for the treatment of cardiovascular diseases. Apoptosis, or the programmed cell death of VSMCs, which occurs in the pathogenesis and progression of vascular proliferative disorders, such as atherosclerosis and restenosis, often represents a critical feature of blood vessel remodeling [4]. In addition, neointima development and lesion growth in VSMCs seem to be restrained by late apoptosis [5]. The regulated balance between the death and survival signals perceived by a cell is used to control the initiation of apoptosis [6]. Because cell apoptosis can inhibit the proliferation of VSMCs, inducing apoptosis may provide a pharmacological basis for treating proliferative cardiovascular disorders.

Increased reactive oxygen species (ROS) production is known to play a vital role in VSMC proliferation and apoptosis and leads to the development of atherosclerosis. The apoptosis of VSMCs caused by enhanced ROS production affects the progression of atherosclerotic lesions and may induce plaque rupture [7]. ROS are small, extremely reactive molecules because of their unpaired valence electrons. There are several intracellular ROS producers, including 2 main manufacturers, the mitochondria and nicotinamide adenine dinucleotide phosphate (NADPH) oxidase. A rapidly expanding body of experimental evidence gathered since the first identification of VSMCs implicates that NADPH oxidase (Nox) in vascular cells is the underlying cause of oxidative stress in various cardiovascular diseases [8]. Nox is a complex composed of membrane-bound (p22phox and Nox1-4) and cytoplasmic (Rac, p47phox, and p67phox) subunits. When it is activated, cytoplasmic subunits connect with their membrane-bound counterparts and generate an active complex that oxidizes NADPH, leading to the production of ROS [9]. The Nox-dependent production of ROS is thought to be a crucial regulator of smooth muscle cell viability and is believed to be linked to the development and severity of human atherosclerotic lesions [10].

**Figure 1:**
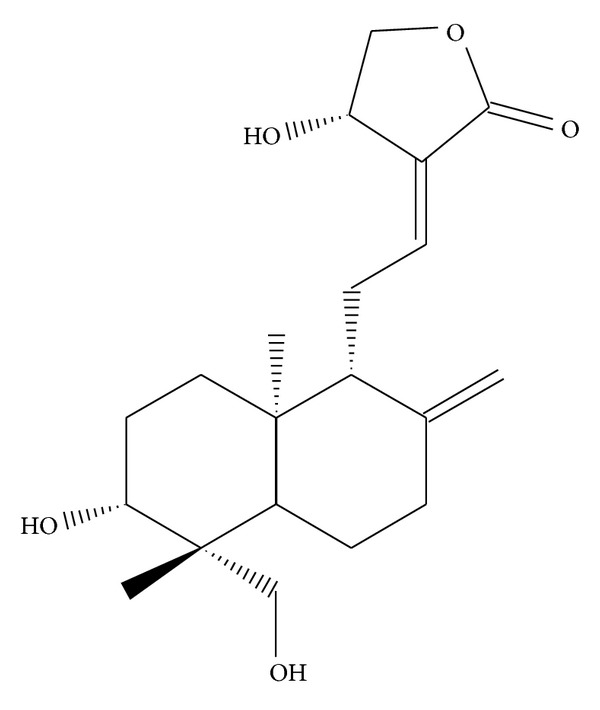
Chemical structure of andrographolide (Andro).

Andrographolide (Figure 1), a novel nuclear factor-*κ*B (NF-*κ*B) inhibitor, is the most active and critical constituent isolated from the leaves of *Andrographis paniculata* [11]. *A. paniculata* has long been used as a herbal medicine to prevent and treat upper respiratory tract infections, diarrhea, rheumatoid arthritis, and laryngitis in Asia and Scandinavia [11, 12]. Recent studies have indicated that andrographolide inhibits tumor growth by inducing cell cycle arrest [13, 14] or apoptosis [15, 16] in various types of cancer cells. Recently, our previous study confirmed that andrographolide enhances NF-*κ*B subunit p65 Ser536 dephosphorylation through neutral sphingomyelinase (nSMase)-mediated ceramide formation in VSMCs [17], involving an increase in cyclic GMP/PKG, followed by the inhibition of the p38MAPK/HO^−^-NF-*κ*B-ERK2 cascade in activated platelets [18, 19]. However, andrographolide has demonstrated antiproliferative and apoptotic effects on various types of cancer cells, whether it induces apoptosis in VSMCs is not known. Furthermore, ROS appear to mediate the apoptosis-inducing activity of andrographolide, but the source of ROS formation in andrographolide-induced apoptosis remains unclear. In the present study, by considering the pivotal role of abnormal VSMC proliferation in the development of atherosclerosis and restenosis, we examined the detailed cellular signaling events associated with andrographolide-induced VSMC apoptosis.

## 2. Materials and Methods

### 2.1. Materials

Dulbecco's modified Eagle's medium (DMEM), trypsin (0.25%), L-glutamine, penicillin/streptomycin, and fetal bovine serum (FBS) were purchased from Gibco (Gaithersburg, MD, USA). Andrographolide (≥98%), 3-(4,5-dimethylthiazol-2-yl)-2,5-diphenyltetrazolium bromide (MTT), N-acetyl-L-cysteine (NAC), diphenyleneiodonium chloride (DPI), 2,7-dichlorofluorescein diacetate (DCF-DA), and dimethyl sulfoxide (DMSO) were obtained from Sigma-Aldrich (St. Louis, MO, USA). The 3-O-methyl-sphingomyelin (3-OMS) was purchased from Biomol (Plymouth Meeting, PA, USA). Anti-caspase-3 monoclonal antibodies (mAbs) and anti-Bax polyclonal antibody (pAb) were obtained from cell signaling (Beverly, MA, USA); the anti-phospho-p47phox serine359 pAb was acquired from Abcam (Cambridge, MA, USA); the anti-*α*-tubulin mAb was obtained from NeoMarkers (Fremont, CA, USA). The hybond-P polyvinylidene difluoride (PVDF) membrane, enhanced chemiluminescence (ECL) Western blotting detection reagent and analysis system, horseradish peroxidase (HRP)-conjugated donkey anti-rabbit immunoglobulin G (IgG), and sheep anti-mouse IgG were acquired from Amersham (Buckinghamshire, UK). Andrographolide was dissolved in 0.1% dimethyl sulfoxide (DMSO) and stored at 4°C until used.

### 2.2. Rat Aortic Smooth Muscle Cell Primary Culture

The male Wistar rats used in this study were purchased from BioLASCO (Taipei, Taiwan). The VSMCs were enzymatically dispersed from the male Wistar rats (250–300 g). Thoracic aortas from the Wistar rats were removed and stripped of endothelium and adventitia. The VSMCs were obtained using a modification of the combined collagenase and elastase digestion method [20]. These cells were grown in DMEM supplemented with 20 mM HEPES, 10% FBS, 1% penicillin/streptomycin, and 2 mM glutamine at 37°C in a humidified atmosphere of 5% CO_2_. The growth medium was changed every 2-3 days until the cells reached confluence. The growth medium was removed, and the monolayer was rinsed with phosphate-buffered saline (PBS). A trypsin-EDTA solution was added, and the monolayer was incubated at 37°C for 2 min. The culture dishes were observed under a phase-contrast microscope until the cells detached. The cells were removed using 10 mL of DMEM and centrifuged at 900 rpm for 7 min. The pellet was resuspended in DMEM in a culture dish, and cells from Passages 4–8 were used in all experiments. The primary cultured rat aortic VSMCs showed the “hills and valleys” pattern, and the expression of *α*-smooth muscle actin was confirmed (data not shown). All protocols were approved by the Taipei Medical University Animal Care and Use Committee.

### 2.3. Cell Viability Assay

The VSMCs (2 × 10^4^ cells/well) were seeded on 24-well plates and cultured in DMEM containing 10% FBS for 24 h. The VSMCs were pretreated with NAC (1 mM) before being treated with andrographolide (50 *μ*M) for 48 h. The cell number was measured using a colorimetric assay based on the ability of mitochondria in viable cells to reduce MTT as previously described [21]. The cell number index was calculated as the absorbance of treated cells/control cells × 100%.

### 2.4. Measurement of Intracellular ROS

The VSMCs (5 × 10^5^ cells/dish) were loaded with DCF-DA (20 *μ*M) for 20 min and then treated following the experimental design. These cells were washed with PBS before trypsinization. The levels of intracellular ROS were detected using Coulter Epics XL flow cytometry (Beckman Coulter, Miami, FL, USA). Data were collected from 10,000 cells per experimental group. All experiments were repeated at least 4 times to ensure reproducibility.

### 2.5. Immunoblot Analysis

Immunoblot analyses were performed as described previously [20]. Briefly, the VSMCs (5 × 10^5^ cells/dish) were treated as the experimental design. After the experimental period, the proteins were extracted using a lysis buffer. Lysates were centrifuged, the supernatant protein (50 *μ*g) was collected and subjected to sodium dodecyl sulfate-polyacrylamide gel electrophoresis (SDS-PAGE), and the separated proteins were electrophoretically transferred onto 0.45 *μ*m PVDF membranes by using semidry transfer (Bio-Rad, Hercules, CA, USA). The blots were blocked with TBST (10 mM Tris-base, 100 mM NaCl, and 0.01% Tween 20) containing 5% bovine serum albumin for 1 h and then probed with various primary antibodies. The membranes were incubated with HRP-linked anti-mouse IgG or anti-rabbit IgG (diluted 1 : 3000 in TBST) for 1 h. The immunoreactive bands were detected using an ECL system. Bar graphs depict the ratios of quantitative results obtained by scanning the reactive bands and quantifying the optical density by using video densitometry (Bio-Profil; Biolight Windows application version 2000.01; Vilber Lourmat, France).

### 2.6. Transfection and Luciferase Reporter Assays

The cells were transfected with PG13-luc and *Renilla*-luc plasmids using the Turbofect reagent. The treated and untreated cells were harvested, and the luciferase activity level was determined using the Dual-Glo luciferase assay system kit. The luciferase activity level was normalized based on the *Renilla* luciferase activity level. The level of luciferase activity was quantified as the ratio of the activity of cells treated with andrographolide to that of the untreated control cells.

### 2.7. Statistical Analysis

The experimental results are expressed as the means ± standard error and are accompanied by the number of observations. Data were assessed using an analysis of variance. If an analysis indicated significant differences among the group means, then each group was compared with the others using the Newman-Keuls method. Values of *P* < 0.05 were considered statistically significant.

## 3. Results

### 3.1. The Role of ROS in Andrographolide-Reduced Cell Viability in Rat VSMCs

**Figure 2:**
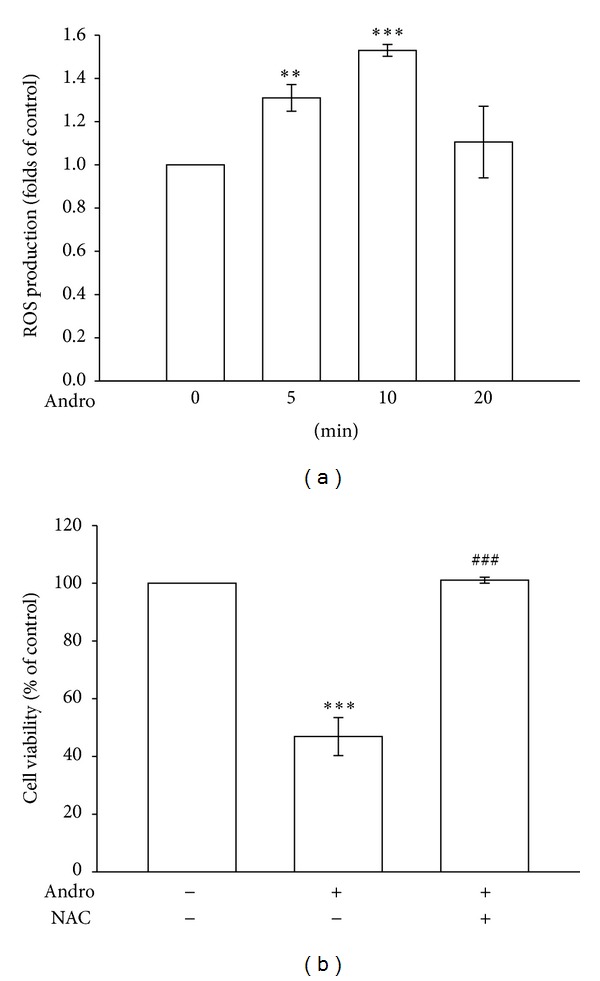
The role of ROS in andrographolide-reduced cell viability in rat VSMCs. (a) Rat VSMCs were treated with 50 *μ*M andrographolide for the indicated periods. Cells were harvested, and the formation of ROS was examined using flow cytometric analysis of DCF-DA-stained cells, as described in Section 2. (b) Cells were pretreated with a vehicle or 1 mM NAC for 30 min before being treated with 50 *μ*M andrographolide for 48 h; cell viability was subsequently determined using an MTT assay. The results shown are representative of 4 independent experiments. The data are presented as the mean ± SEM (error bars: **P < 0.01 and ***P < 0.001, compared with the control group, and ^###^ *P* < 0.001, compared with the group treated only with andrographolide).

We previously determined that andrographolide resulted in loss of cell viability in a concentration-dependent manner by using an MTT assay (unpublished data). However, the detailed mechanism involved in this phenomenon remains unclear. ROS formation is known to play a crucial role in cell apoptosis [7]. Therefore, we investigated the role of ROS in andrographolide-induced VSMC death.  Figure 2(a) shows that treatment with 50 *μ*M andrographolide significantly induced ROS formation 1.5 ± 0.0-fold at 10 min compared with the control group (*P* < 0.001, *n* = 4). We subsequently preincubated with NAC (1 mM) an ROS scavenger, in andrographolide-treated VSMCs.  Figure 2(b) shows that treatment with andrographolide reduced the cell viability of rat VSMCs to 46.9 ± 6.6% compared with the control group (*P* < 0.001, *n* = 4), whereas pretreatment with 1 mM NAC significantly reversed the andrographolide-induced reduction in VSMC viability (*P* < 0.001, *n* = 4). Taken together, these data suggest that the reduction of cell viability in andrographolide-treated rat VSMCs was related to the cellular redox state, possibly as a consequence of ROS formation.

### 3.2. Nox-Mediated Redox Signaling in Andrographolide-Induced ROS Formation

**Figure 3:**
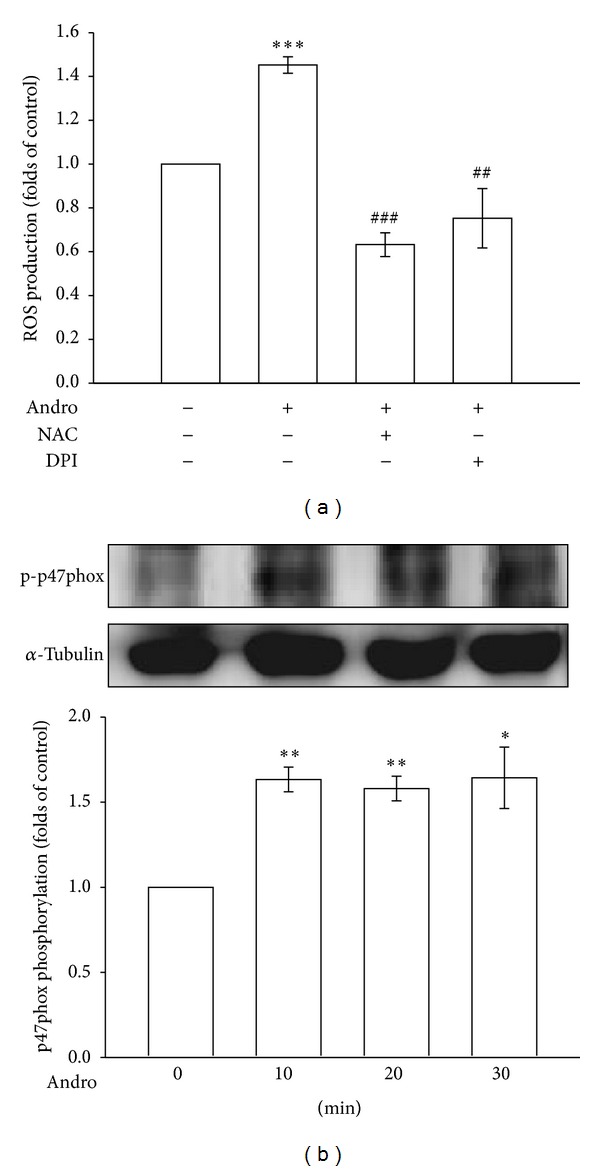
Nox-mediated redox signaling in andrographolide-induced ROS formation. (a) Cells were pretreated with a vehicle, 1 mM NAC, or 10 *μ*M DPI for 30 min before being treated with 50 *μ*M andrographolide for 10 min, and the production of ROS was examined using flow cytometric analysis of DCF-DA-stained cells, as described in Section 2. (b) Cells were treated with 50 *μ*M andrographolide for the indicated periods. Cells were harvested, and the phosphorylation of p47phox was examined using immunoblotting. The data are presented as the mean ± SEM (error bars: *P < 0.05, **P < 0.01, and ***P < 0.001, compared with the control group, and ^##^ *P* < 0.01 and ^###^ *P* < 0.001, compared with the group treated only with andrographolide).

Coronary artery restenosis, a frequent complication of angioplasty, is accompanied by an increase in Nox-generated ROS production [22]. Therefore, we investigated the involvement of Nox-mediated signaling in andrographolide-induced ROS formation. In Figure 3(a), pretreatment with NAC (1 mM) or DPI (10 *μ*M), a Nox inhibitor, significantly suppressed andrographolide-induced ROS formation. Furthermore, growing evidence has suggested that the activation of the Nox subunit p47phox is required for ROS production in vascular cells [23]. We subsequently determined whether Nox subunit activation is required for andrographolide-induced ROS production in rat VSMCs. As shown in Figure 3(b), serine359 phosphorylation of the p47phox subunits was significantly increased 1.6 ± 0.1-fold after andrographolide stimulation for 10 min compared with the control group (*P* < 0.01, *n* = 3). These results suggest that phosphorylation of the Nox subunit p47phox mediates ROS formation in rat VSMCs treated with andrographolide.

### 3.3. Effects of ROS Scavengers on Andrographolide-Stimulated p53 Activation, Bax, and Active Caspase-3 Expression in Rat VSMCs

**Figure 4:**
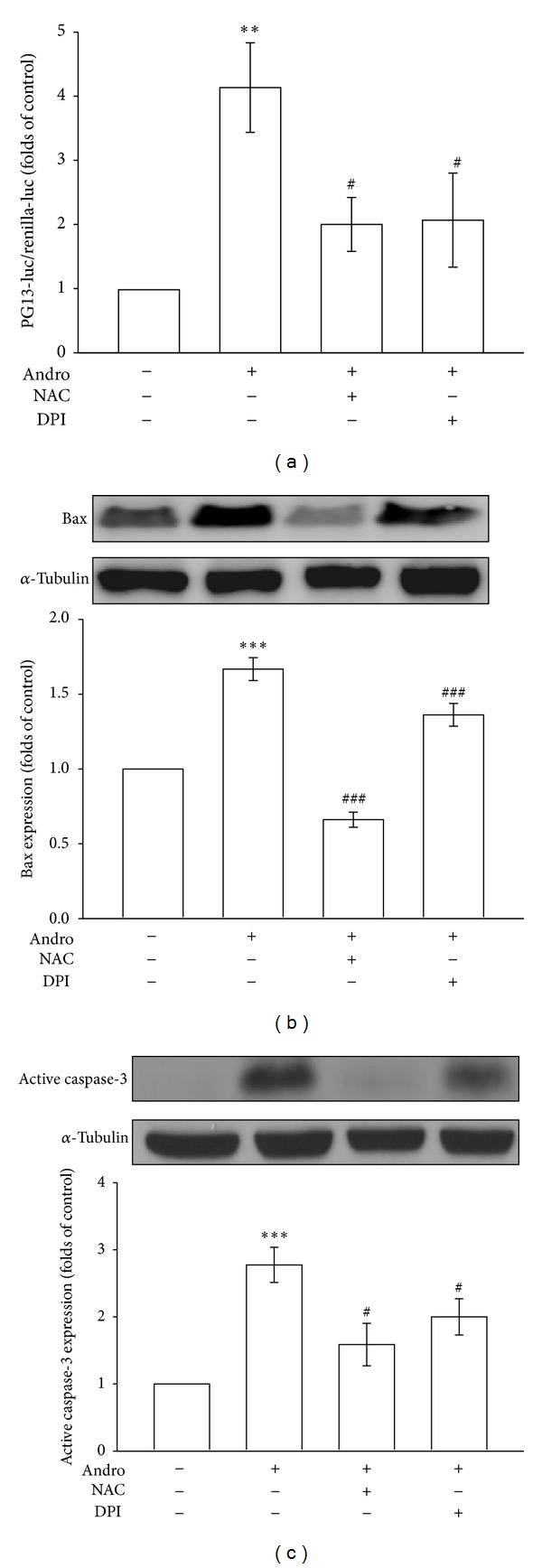
Effects of ROS scavengers on andrographolide-stimulated p53 activation, Bax, and active caspase-3 expression in rat VSMCs. (a) Cells were transiently transfected with PG-13-luc and *Renilla*-luc for 48 h. After transfection, the cells were pretreated with a vehicle, 1 mM NAC, or 10 *μ*M DPI for 30 min before being treated with 50 *μ*M andrographolide for another 24 h. A PG13-luciferase assay was subsequently conducted. Cells were pretreated with a vehicle, 1 mM NAC, or 10 *μ*M DPI for 30 min before being treated with 50 *μ*M andrographolide for 48 hr, and the expression of Bax (b) and active caspase-3 (c) was examined using immunoblotting. The data are presented as the mean ± SEM (error bars: **P < 0.01 and ***P < 0.001, compared with the control group, and ^#^ *P* < 0.05, ^##^ *P* < 0.01, and ^###^ *P* < 0.001, compared with the group treated only with andrographolide).

It is known that oxidative stress triggers the activation and nuclear translocation of p53 [24], and p53-induced apoptosis involves the generation of ROS [25]. Therefore, we used a PGl3-Luc reporter construct that contained a p53 DNA-binding site linked to a basal promoter that controls the expression of a luciferase reporter gene [26] to examine whether p53 transactivation increases in cells exposed to andrographolide. As shown in Figure 4(a), cells treated with 50 *μ*M andrographolide for 24 h exhibited a 4.2 ± 0.7-fold increase in PG13-luciferase activity level compared with the control group (*P* < 0.01, *n* = 5). Pretreating cells with NAC or DPI apparently inhibited the andrographolide-induced increase in PG13-luciferase activity 68.8% and 65.6%, respectively (*n* = 5) (Figure 4(a)).

It has been suggested that the activation of p53 regulated and promoted discrete steps of the apoptosis cascade such as the upregulation of Bax genes [27] and the overexpression of Bax accelerates apoptotic death through interaction with components of the permeability transition pore complex, causing the opening and rupture of its outer mitochondrial membrane [28]. As shown in Figure 4(b), treatment with 50 *μ*M andrographolide for 48 h significantly induced Bax expression 1.7 ± 0.1-fold compared with the control group (*P* < 0.001, *n* = 3), whereas pretreatment with NAC or DPI significantly inhibited andrographolide-induced reductions in Bax expression 59% and 18%, respectively (*n* = 3). The expression levels of active caspase-3, an apoptotic-pathway-related proapoptotic protein, were subsequently determined in andrographolide-treated VSMCs. As shown in Figure 4(c), treatment with andrographolide for 48 h significantly increased the levels of active caspase-3 2.8 ± 0.3-fold at the concentration of 50 *μ*M compared with the control group (*P* < 0.001, *n* = 4), whereas pretreatment with NAC or DPI significantly inhibited andrographolide-induced reductions in active caspase-3 expression by 43% and 29%, respectively (*n* = 4). These results suggest that Nox-mediated redox signaling induces p53 activation as well as Bax and active caspase-3 expression in andrographolide-treated rat VSMCs.

### 3.4. The Role of Ceramide Signaling in Andrographolide-Induced p47phox Phosphorylation, Bax, and Active Caspase-3 Expression in Rat VSMCs

**Figure 5:**
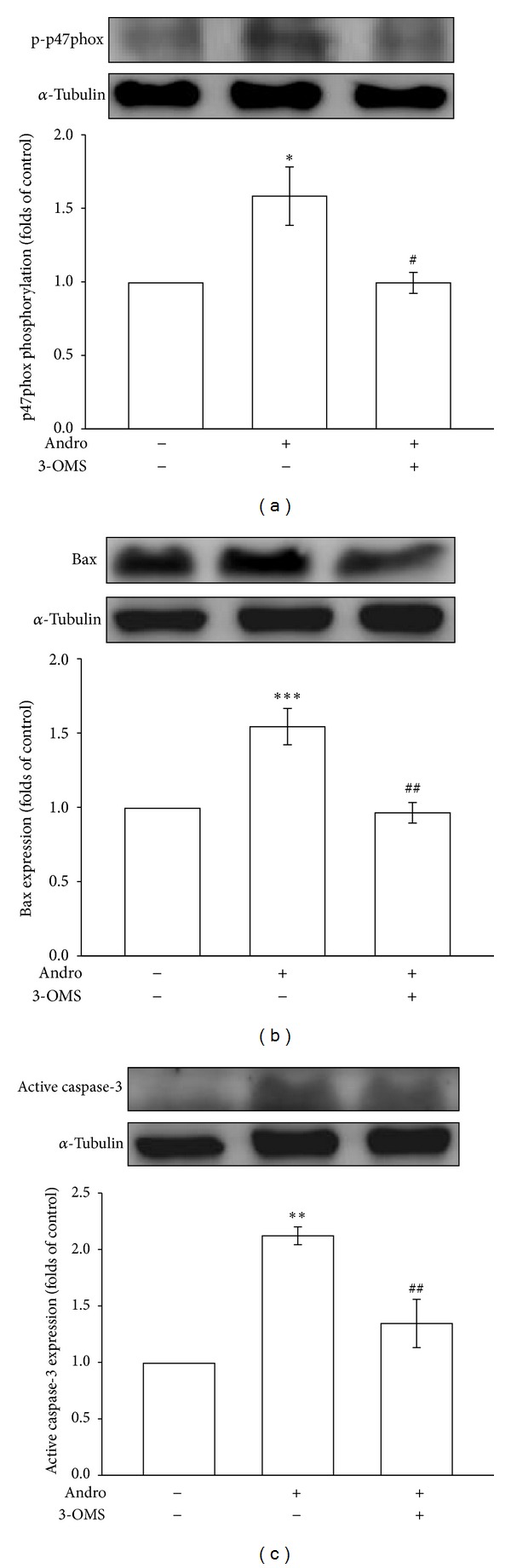
The role of ceramide signaling in andrographolide-induced p47phox phosphorylation, Bax, and active caspase-3 expression in rat VSMCs. Cells were pretreated with a vehicle or 30 *μ*M 3-OMS for 30 min before being treated with 50 *μ*M andrographolide for 10 min (a), or 48 h (b and c). The extent of p47phox phosphorylation (a), Bax (b), or active caspase-3 expression (c) was examined. The data are presented as the mean ± SEM (error bars: *P < 0.05, **P < 0.01, and ***P < 0.001, compared with the control group, and ^##^ *P* < 0.01 and ^###^ *P* < 0.001, compared with the group treated only with andrographolide).

The precise mechanism involved in the andrographolide-induced phosphorylation of p47phox in rat VSMCs remains unclear. A previous study reported ceramide to be a critical signaling molecule that mediates the activation of Nox in various cells [29]. In addition, we demonstrated that andrographolide can activate the nSMase-ceramide cascade in rat VSMCs, and andrographolide-induced ceramide formation was markedly attenuated by 3-OMS, an nSMase inhibitor [17]. As shown in Figure 5(a), pretreatment with 3-OMS (30 *μ*M) for 30 min significantly inhibited andrographolide-induced p47phox phosphorylation 38% (*P* < 0.05, *n* = 3). Pretreatment with 3-OMS also significantly diminished andrographolide-induced Bax and active caspase-3 expression in rat VSMCs (Figures 5(b) and 5(c)).

## 4. Discussion

VSMCs represent a moving component of the vasculature and constitute the medial layer of blood vessels. VSMCs following pathological stimuli can adopt a “de-differentiated” phenotype or undergo hypertrophy and synthesize excess extracellular matrix and inflammatory cytokines, which divide and migrate toward the intima. The abnormal proliferation and reduced apoptosis can lead to excessive accumulation of VSMCs in the intima and media of atherosclerotic lesions involved [30]. A variation in the balance between the proliferation and apoptosis of VSMCs is considered to play a vital role in the development of atherosclerosis and cardiovascular diseases [31, 32]. Thus, maintaining the alteration between the proliferation and apoptosis of VSMCs has been proposed as an effective therapeutic method for preventing and treating vascular diseases, including atherosclerosis [33]. Apoptosis (programmed cell death) in a wide range of physiological settings is to remove discarded cells [34]. Recent studies have indicated that andrographolide inhibits tumor growth by inducing cell cycle arrest [13, 14] or apoptosis [15, 16] in various types of cancer cells. In the present study, andrographolide was also observed to induce apoptosis in rat VSMCs, whereas no cytotoxic effect was observed (data not show), suggesting that andrographolide may be a potential therapeutic agent in VSMC-proliferation-related diseases. 

The net balance between proliferation, apoptosis, and necrosis determines the extent of cell growth. A growing body of evidence now suggests that ROS play a role in both cellular necrosis and apoptosis [35]. Andrographolide was reported to induce ROS and caspase-dependent apoptosis in lymphoma cell lines and in primary tumor samples [36]. Therefore, we hypothesized that andrographolide might cause apoptosis in rat VSMCs through mechanisms that involve cellular redox systems. We determined that the effects of andrographolide were concentration related and accompanied by ROS generation (Figures 2 and 3(a)).

The proapoptotic protein Bax is known to cause apoptosis by disrupting mitochondrial integrity [37]. Yang et al. demonstrated that andrographolide induces the expression of Bax, activates caspases, and stimulates apoptosis in lymphoma cells [36]. Activation of p53 is known to increase the expression of Bax in response to selected stress signals [38]. A recent study observed that andrographolide can activate p53 through ROS-dependent to TRAIL-induced apoptosis in cancer cells [39]. Among ROS, O_2_^−^ is highly reactive and short lived and can spontaneously or enzymatically dismutate to a second signaling intermediate, H_2_O_2_, through superoxide dismutase. Although the production of O_2_^−^ contributes to the primary biological activity of Nox, much of the signaling is mediated by the dismutation product H_2_O_2_. H_2_O_2_ is more stable than O_2_^−^ and is capable of crossing biological membranes and inducing nucleus DNA damage to cause a p53-dependent pathway [40, 41]. In the present study, andrographolide-induced p53 activation, Bax, and active caspase-3 expressions were significantly diminished by treatment with NAC and DPI. These data indicate the involvement of the ROS-mediated p53-Bax-caspase apoptotic pathway in andrographolide-induced VSMC apoptosis (Figure 4).

Noxs are multiprotein complexes of various compositions depending on the cell type. This enzyme, originally described in phagocytes, consists of 2 membrane-bound subunits (p22phox and Nox2) and 3 cytosolic subunits, such as p47phox, p67phox, and Rac1 (nonphagocytes) or Rac2 (phagocytes), which are recruited upon activation to the membrane-bound Nox/p22phox complex. VSMCs contain several sources of ROS, among which the Nox1 and Nox2 are predominant. Barry-Lane et al. have suggested that p47phox is the only subunit that is used specifically by Nox2 and by Nox1 expressed in VSMCs [42]. Furthermore, p47phox for oxidase activation requires a phosphorylated serine at position 359 that is absolutely required for oxidase activity and must be phosphorylated to allow translocation [43]. A functional role for p47phox has also been shown using VSMCs from p47phox knockout mice, in which the agonist stimulation of ROS was reduced [44, 45]. In the present study, we observed that DPI, a Nox inhibitor, significantly restored ROS formation and apoptosis-inducing activity in andrographolide-treated rat VSMCs, and the incubation of andrographolide apparently increased the phosphorylation of p47phox, a Nox subunit. These data collectively indicate that Nox-mediated redox signaling plays a crucial role in rat VSMCs treated with andrographolide. However, the precise mechanism involved in the andrographolide-induced phosphorylation of p47phox in rat VSMCs remains unclear.

Ceramide, the central core lipid in the metabolism of sphingolipids, is produced through hydrolysis of complex sphingolipids, such as sphingomyelin, by mammalian SMases or through the acylation of a long-chain sphingoid base (sphingosine) in a *de novo* biosynthetic pathway. The SMases and its role in ceramide metabolism are the most extensively studied. Recent studies have demonstrated that ceramide increased in endothelial cells exposed to death factors, including tumor necrosis factor *α*, interleukin 2, and endostatin, and in ischemic reperfused myocardium [46, 47]. In these studies, the ceramide signaling pathway has been confirmed to be involved in the activation of Nox and consequent O_2_^−^ production [48, 49]. Ceramide also enhances ROS formation in mammalian cells by straightly raising the permeability of mitochondrial membranes to cytochrome c [50] and restraining the isolated mitochondria complex III [51]. In addition, the accumulation of ceramides caused by the activation of SMases has been observed in response to various stimuli, such as oxidants and heat stress [52, 53]. On the other hand, our previous study found that andrographolide can directly enhance ceramide level in VSMCs, and this phenomenon was markedly attenuated by 3-OMS [17]. In the present study, we observed that 3-OMS apparently abolished andrographolide-induced p47phox phosphorylation, Bax, and active caspase-3 expression in rat VSMCs (Figure 5). Taken together, these results indicate that andrographolide increases the turnover of sphingomyelin in rat VSMCs, and this lipid-signaling pathway may mediate the action of the andrographolide-induced activation of Nox, resulting in O_2_^−^ production and apoptosis-inducing activity in rat VSMCs. 

**Figure 6:**
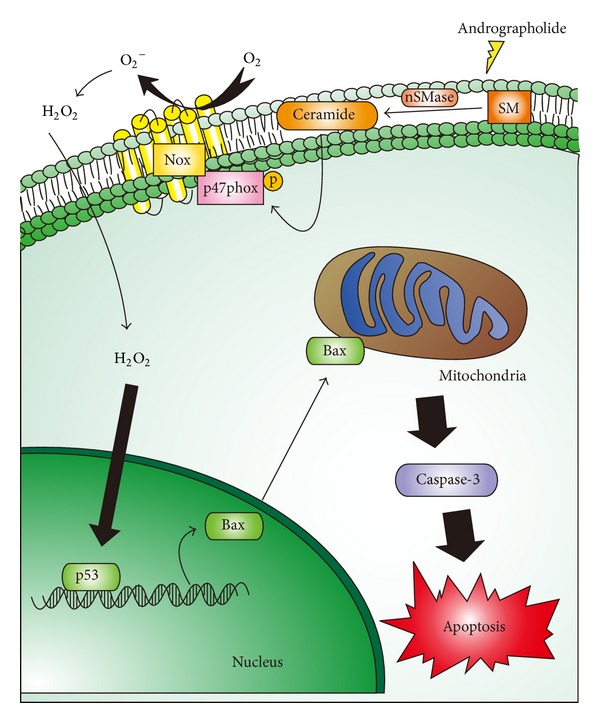
Hypothetical scheme of the signal pathways in andrographolide-induced rat VSMC apoptosis. Andrographolide stimulated the ceramide-mediated signal events, resulting in the activation of the p47phox-ROS cascade, ultimately stimulating active caspase-3 expression and VSMC apoptosis. Nox produces superoxide (O_2_^−^), followed by the induction of H_2_O_2_. H_2_O_2_ is capable of inducing DNA damage to cause p53 activation, which can lead Bax and active caspase-3 expression. nSMase: neutral sphingomyelinase; SM: sphingomyelinase; Nox: NADPH oxidase.

In the present study, we showed that andrographolide, the active component of the plant *A. paniculata*, has the ability to reduce cell viability in rat VSMCs. The in-depth mechanism of its apoptosis-inducing activity is related to the Nox-mediated redox signaling of cells, because this signaling is blocked by NAC and DPI. In addition, this is the first study to indicate the role of the ceramide-p47phox signaling pathway in andrographolide-induced ROS-mediated cell apoptosis (Figure 6). In conclusion, we showed that the ceramide-p47phox-ROS signaling cascade may contribute to andrographolide-induced VSMC apoptosis. Using this novel natural lactone diterpenoid as a therapeutic strategy for cardiovascular disorders involving VSMC proliferation and atherogenesis warrants further preclinical and clinical investigation.
